# The Effect of Blood Pressure Variability on Coronary Atherosclerosis Plaques

**DOI:** 10.3389/fcvm.2022.803810

**Published:** 2022-03-15

**Authors:** Yue Liu, Xing Luo, Haibo Jia, Bo Yu

**Affiliations:** ^1^Department of Cardiology, 2nd Affiliated Hospital of Harbin Medical University, Harbin, China; ^2^Key Laboratory of Myocardial Ischemia, Ministry of Education, Harbin Medical University, Harbin, China

**Keywords:** blood pressure variability, coronary heart disease, plaque rupture, plaque erosion, inflammation, hemodynamics, smooth muscle cell, endothelial cell

## Abstract

Hypertension is one of the most important risk factors for coronary heart disease (CHD). The regulation of blood pressure plays a significant role in the development and prognosis of CHD. Blood pressure variability (BPV) refers to the degree of fluctuation of blood pressure over a period of time and is an important indicator of blood pressure stability. Blood pressure fluctuations are complex physiological phenomena, being affected by physiological and pharmacological effects and regulated by behavioral, environmental, hydrodynamic, and neural factors. According to the different time periods for measuring BPV, it can be divided into very short-term, short-term, mid-term, and long-term. Multiple cardiovascular disease animal models and clinical experiments have consistently indicated that abnormal BPV is closely related to coronary events and is a risk factor for CHD independently of average blood pressure. Thrombosis secondary to plaque rupture (PR) or plaque erosion can cause varying blood flow impairment, which is the main pathological basis of CHD. Plaque morphology and composition can influence the clinical outcome, treatment, and prognosis of patients with CHD. Research has shown that PR is more easily induced by hypertension. After adjusting for the traditional factors associated with plaque development, in recent years, some new discoveries have been made on the influence of abnormal BPV on the morphology and composition of coronary plaques and related mechanisms, including inflammation and hemodynamics. This article reviews the impact of BPV on coronary plaques and their related mechanisms, with a view to prevent the occurrence and development of CHD by controlling BPV and to provide new prevention and treatment strategies for the clinical treatment of abnormal blood pressure.

## Introduction

Since the middle of the last century, cardiovascular diseases (CVDs) have been the leading cause of human death worldwide, accounting for approximately half of all deaths (1), of which acute coronary syndrome (ACS) is the most important cause (2). Plaque rupture (PR) and plaque erosion (PE), the two major pathological mechanisms of ACS, can be accurately identified using intracoronary imaging (3). Hypertension, as a critical independent coronary risk factor, has been confirmed to be closely associated with PR (4, 5). To date, the effect of hypertension on CVDs and coronary plaque morphology has been given the utmost attention in pathological, clinical, and imaging studies. However, when a battery of methods to improve the control rate of hypertension was taken, the risk of CVDs caused by hypertension did not decline, or even increased. For the sake of exploring the reason behind this fact, researchers have repeatedly found that blood pressure will fluctuate under the influence of multiple factors (e.g., neuroregulation, humoral factors, environmental factors), thus forming the concept of blood pressure variability (BPV) (6). Increments in BPV can lead to a plethora of detrimental effects, such as accelerating the progression of coronary plaques and the degree of arterial stiffness, promoting vascular endothelial injury, as well as activation of inflammatory factors, leading to target organ damage and major adverse cardiac events (MACE) (6–8). Accumulating evidence indicates that BPV is closely related to CVD risk. In addition, compared with the mean blood pressure, BPV is more relevant to coronary artery disease and its prognosis (9, 10). Therefore, BPV can be regarded as a relevant index which can provide a therapeutic target for the prevention and treatment of CVDs. To minimize the residual risks after the treatment of hypertension, it is important to clarify the effects of BPV on coronary plaque and its mechanism of action.

In this review, we will offer an overview of BPV and summarize its effects on coronary events as well as on coronary atherosclerosis plaque development and progression. Furthermore, the mechanisms of the effects of BPV on coronary atherosclerotic plaque formation will be addressed.

## Blood Pressure Variability

### Various Types

The term BPV was coined ~50 years ago to describe the phenomenon of fluctuations in blood pressure. Whether this variability increases or decreases will affect the body's self-regulation (11, 12). Clinically, BPV is usually divided into four categories according to the length of monitoring time: very short-term, short-term, mid-term, and long-term. The BPV present in each cardiac cycle is the very-short-term BPV, which can be obtained by non-invasive finger blood pressure monitoring or invasive artery cannulation techniques. However, considering the practicality and economy of measuring methods, their clinical applications are limited (13). Generally, short-term refers to blood pressure that varies within 24 h, which can be mainly affected by neural, humoral, and emotional factors. Its application is not only helpful in detecting masked hypertension but is also a reliable predictor of target organ damage and cardio-cerebrovascular diseases (6). The most reliable method for monitoring short-term BPV is 24 h ambulatory blood pressure monitoring (ABPM), which can automatically monitor blood pressure every 15–30 min throughout the day. The most common quantitative indicators of short-term BPV include standard deviation (SD), coefficient of variation (CoV), and average real variability (ARV), among which the latter possesses a stronger predictive ability since it can dampen the effect of average blood pressure on BPV (8, 14).

There is no clear or uniform clinical definition of mid-term or long-term BPV. In general, the mid-term represents daily blood pressure fluctuations, which can be measured by ABPM, office blood pressure monitoring (OBPM), and home blood pressure monitoring (HBPM) (8). Among them, ABPM often requires at least 48 h, which is beyond the tolerance of patients. Therefore, until the use of new wearable devices is deemed safe and effective in clinical studies, OBPM and HBPM remain more commonly used (15). Previous studies have found that mid-term BPV is susceptible to the frequency and duration of blood pressure measurements (16). Therefore, the question of the opportunity to monitor the mid-term BPV is still under debate. Finally, long-term BPV reflects fluctuations across follow-up appointments, months, seasons, or even years and has been proven to be advantageous in predicting coronary events, strokes, and other adverse cardiovascular events (8, 17). Compared with intrinsic factors, environmental factors are more likely to affect long-term BPV (18). Owing to the high repeatability and compliance of blood pressure management models based on HBPM, so it is quite popular in clinical practice, followed by OBPM. However, although ABPM can reduce the impact of white coat hypertension, thereby improving accuracy, its use is greatly restricted by patient tolerance. Unlike mid-term BPV, long-term BPV is only positively correlated with the frequency of follow-up (19). In addition, the indices of measurement for the long term are the same as those for the mid-term (SD and CoV).

### Influencing Factors

The mechanisms that regulate BPV are extremely complex. Physically, BPV is mainly affected by the regulation of the autonomic nervous system and baroreceptors. Through tests in mice, Zeng et al. discovered that BPV increased after simultaneous ablation of Piezo1 and Piezo2, which are mechanosensitive ion channels required for baroreceptor activity (20). Persu et al. performed renal denervation (RDN) in 167 patients with resistant hypertension and compared the blood pressure at baseline and at 6 months after surgery. It was observed that mean office systolic/diastolic blood pressure decreased by 15.4/6.6 mmHg and 24 h weighted SD of systolic/diastolic BPV decreased by 1.18/0.63 mmHg, respectively. Therefore, they suggested that RDN has a considerable effect on the treatment of increased BPV caused by sympathetic nerve excitability (21). In additon to the nervous system, BPV can also be influenced by changes in arterial elasticity, humoral factors (levels of e.g., endothelin-1, insulin, bradykinin), emotional and behavioral factors (anxiety, postural changes, lifestyle), environmental factors (atmospheric pressure, climate change), and drugs (6, 22, 23). To explore the effects of dietary potassium and sodium on BPV, Chang et al. calculated the urinary sodium-to-potassium ratio of 343 hypertensive patients and pointed out that the combination of high-potassium and sodium-restricted diet is more effective in alleviating blood pressure fluctuations than a purely high-potassium or sodium-restricted diet (24). Moreover, different antihypertensive medical therapies can have various degrees of impact on BPV. On one hand, when treated with a single antihypertensive drug, calcium antagonists have obvious advantages in controlling BPV (25). A previous meta-analysis of 1,530 trials showed that calcium antagonists or diuretics have the most significant effects on the control of BPV. In contrast, angiotensin converting enzyme inhibitors (ACEI), angiotensin receptor blockers (ARB), and β-blockers not only cannot effectively control BPV, but also have the risk of increasing BPV (26). To further understand the impact of different kinds of antihypertensive drugs on BPV and explore whether these effects can explain the differences in cardiovascular events and mortality between patients, Mehlum et al. randomly assigned 14 996 patients with hypertension to the valsartan or amlodipine groups and performed a 5-year follow-up. They reported that compared with patients in the valsartan group, visit-to-visit mean blood pressure and systolic BPV were 2.2 and 1.4 mmHg lower in the amlodipine group, respectively. In addition, the probabilities of myocardial infarction and all-cause death in the amlodipine group were reduced by 0.7 and 0.1%, respectively, compared to those in the valsartan group. Hence, they concluded that calcium antagonists not only have a better ability to control BPV but can also reduce the morbidity and mortality of myocardial infarction caused by BPV (27). On the other hand, BPV is more stable when taking a variety of antihypertensive drug treatments, such as CCB/ARB or diuretics/ARB than with monotherapies (28). Parati et al. recruited 4,294 patients with hypertension to compare the effects of telmisartan/amlodipine to the corresponding monotherapy and placebo on 24 h BPV. That study indicated that CCB/ARB therapy is more effective in reducing 24-h BPV than other treatments (29). In addition to traditional antihypertensive drugs, a meta-analysis of 2,381 hypertensive patients indicated that sodium-glucose cotransporter (SGLT)-2 inhibitors also play an active role in stabilizing short-term BPV. In the research, 24 h ABPM was used to evaluate the diurnal blood pressure fluctuations in the SGLT2-2 inhibitors group and the placebo group. The results showed that the diurnal systolic and diastolic blood pressure fluctuations of the SGLT2-2 inhibitors group were 3.62 mmHg and 1.70 mmHg lower than those in the placebo group (30). The above studies indicated that BPV may be affected by the type or combination of antihypertensive drugs. Therefore, in order to improve the protective effects on cardiovascular disease, it is necessary to understand the mechanisms of BPV, the mechanisms of different types of antihypertensive drugs on BPV, as well as the effects of antihypertensive agents and pharmaceutical processes on BPV. Therefore, there is still an urgent requirement for large-scale prospective trials for in-depth exploration.

## The Effects of BPV on Coronary Events

A growing number of trials and clinical data have indicated that BPV is a powerful potential predictor of coronary artery disease. Moreover, the higher the BPV, the higher likelihood of complicated coronary artery disease (31–33). Gosmanova et al. analyzed the data of 2 865 157 American veterans with at least eight follow-up blood pressure records. During a median period of 8 years of follow-up, the highest incidence of coronary heart disease (CHD) was 2.7%. After multivariate adjustment, comparison of the lowest and highest quartiles of visit-to-visit BPV gradually increased the risk of CHD (hazard ratio [HR] 5.92, 95% confidence interval [95% CI] 5.70–6.14) (34). Suchy-Dicey et al. conducted a subgroup analysis of the Cardiovascular Health Study which included 1 642 participants. They found that the higher the long-term BPV, the higher the incidence of myocardial infarction (HR 1.20, 95% CI 1.06–1.36) (35). Muntner et al. performed a subgroup analysis of Antihypertensive and Lipid-Lowering Treatment to prevent Heart Attack Trail and demonstrated that the highest quintile of visit-to-visit BPV is more likely to develop fatal or non-fatal CHD (HR 1.30, 95% CI 1.06–1.59) (36). The Valsartan Antihypertensive Long-term Use Evaluation subgroup trial conducted by Mehlum et al. showed a strong relationship between long-term BPV and myocardial infarction (HR 3.2, 95% CI 2.3–4.3) (17). A *post-hoc* analysis of two large-scale studies, the Anglo-Scandinavian Cardiac Outcomes Trail Blood Pressure Lowering Arm and the Medical Research Council showed that when amlodipine was applied to stabilize blood pressure oscillations, the incidence of coronary events also decreased (25). The predictive ability of BPV for coronary artery disease has been well-established through numerous large-scale randomized clinical trials. Furthermore, in recent years, several studies have further analyzed which BPV has the best predictive power for coronary events. Dai et al. compared the mortality ratios of CVDs between a short-term BPV group containing 24,004 patients and a long-term BPV group containing 30,506 patients. They reported that the correlation between short-term BPV and CVD mortality was stronger than that of long-term BPV (37). Similarly, Zheng et al. followed 42,154 subjects for an average of 12.5 years and found that compared with long-term BPV, short-term BPV was slightly better in predicting the incidence of myocardial infarction or the mortality of MACE (38). However, other experimental studies showed diametrically opposing results. A meta-analysis showed that long- and short-term BPV had the same correlation with all-cause mortality. In addition, long-term systolic BPV was associated with CHD (HR 1.10, 95% CI 1.04–1.16), but the relationship between short-term BPV and CHD was negligible (8). Mallamaci et al. studied the effects of short-term and long-term BPV on CVD in 402 patients with chronic kidney disease. That study determined that for every 5 mmHg increase in SD of long-term systolic BPV, the HR (95% CI) of CVDs increased 1.24 (1.01–1.51), compared to 0.92 (0.68–1.25) in short-term BPV (39). To date, there is still controversy regarding which period of BPV is a better predictive factor. The methods used to monitor BPV vary, moreover, the main influence mechanisms of diverse BPV are distinct. And perhaps there may be no clear correlation or comparison between the two different types of BPV. Hence, in order to answer this question, further clinical research is required (Table 1).

**Table 1:** Representative studies of the effects of BPV on coronary events.

**References**	**Number in study**	**Purposes**	**Follow up time**	**End point**	**Conclusions**
Gosmanova et al. (34)	2,865,157 American veterans with and without hypertension	To explore the relationship between increased visit-to visit BPV and all-cause mortality, cardiovascular disease and end-stage renal disease.	During a median period of 8 years of follow-up	All-cause mortality, incident CHD, ischemic strokes and end-stage renal disease	Higher visit-to-visit BPV increased the risk of all-cause mortality (HR 1.80, 95%CI 1.78–1.82), incident CHD (HR 5.92, 95%CI 5.70–6.14), ischemic strokes (HR 6.60, 95%CI 6.32–6.89) and end-stage renal disease (HR 10.59, 95%CI 9.02–12.43).
Suchy-Dicey et al. (35)	1,642 participants	To assess the relationship between the long-term systolic BPV and all-cause mortality, incident myocardial infarction and incident stroke.	Over a mean period of 9.9 years of follow-up	All-cause mortality, incident myocardial infarction and incident stroke	Higher long-term systolic BPV had a strong correlation with all-cause mortality (HR 1.13, 95%CI 1.05–1.21) and myocardial infarction (HR 1.20, 95%CI 1.06–1.36), but not stroke.
Muntner et al. (36)	25,814 hypertensive patients	To examine the impact of visit-to-visit BPV on CVDs and mortality.	Over a mean period of 2.7 to 2.9 years of follow-up	Fatal CHD or non-fatal myocardial infarction, all-cause mortality, stroke and heart failure.	Higher visit-to-visit BPV increased the risk of fatal or non-fatal CHD (HR 1.30, 95%CI 1.06–1.59) and all-cause mortality (HR 1.58, 95%CI 1.32–1.90).
Mehlum et al. (17)	13,803 hypertensive patients	To assess if BPV in hypertensive patients at different risk levels can increase the risk of CVDs and death.	During a mean period of 4.2 years of follow-up	Cardiac event and stoke.	Higher visit-to-visit BPV was associated with the increased risk of myocardial infarction (HR 3.2, 95%CI 2.3–4.3), heart failure (HR 3.1, 95%CI 2.2–4.3), cardiovascular event (HR 2.1, 95%CI 1.7–2.4) and stroke (HR 1.9, 95%CI 1.3–2.7).
Rothwell et al. (25)	23,653 hypertensive patients in total, of which 19,257 were from the Anglo-Scandinavian Cardiac Outcomes Trail Blood Pressure Lowering Arm trail, 4,396 were from the Medical Research Council	To investigate whether different kinds of antihypertensive drugs may have additional benefits in reducing adverse vascular events by smoothing blood pressure fluctuations.	6 years	Coronary events and stroke.	Calcium antagonists had obvious advantages in controlling BPV. As blood pressure fluctuations became stable, the incidence of coronary events also decreased.
Dai et al. (37)	24,004 participants in short-term analysis, 30,506 participants in long-term analysis	To compare the impacts of short-term BPV and long-term BPV on the mortality of all-cause and CVDs.	12.5 years	All-cause and CVD mortality	Higher short-term BPV had a greater impact on all-cause and CVD mortality than long-term BPV.
Zheng et al. (38)	19,544 subjects in short-term analysis, 22 610 subjects in long-term analysis	To compare the predictive ability of short-term and long-term BPV on clinical outcomes.	During a median period of 12.5 years of follow-up	MACE, myocardial infarction, CVD death, Stroke	Both short-term BPV and long-term BPV could increase the risk of MACE. In addition, compared with long-term BPV, short-term BPV has a better ability to predict myocardial infarction.
Stevens et al. (8)	A meta-analysis included 36 studies	To explore the impact of various types of BPV on CVDs and mortality.	No follow-up visits	All-cause and CVD mortality and CVD events	All kinds of BPV had correlation with cardiovascular and mortality, but long-term BPV possessed a better predictive ability for CHD (HR 1.10, 95%CI 1.04–1.16) than short-term BPV.
Mallamaci et al. (39)	402 patients with chronic kidney disease	To assess the effects of short-term and long-term BPV on the risk of CVDs in patients with chronic kidney disease.	Over a mean period of 4.8 years of follow-up	All-cause mortality and cardiovascular events	In patients with chronic kidney disease, each 5 mmHg increase in SD of long-term systolic BPV, the HR (95%CI) of CVDs increased 1.24 (1.01–1.51), compared to short-term BPV 0.92 (0.68–1.25).

*CHD, coronary heart disease; BPV, blood pressure variability; CVD, cardiovascular disease; MACE, major adverse cardiac events; HR, hazard ratio; CI, confidence interval; SD, standard deviation*.

Multitudinous sources of evidence suggested that abnormal BPV is not only a potential predictor of coronary artery disease, but also closely associated with the occurrence of heart failure or arrhythmia. Daniel et al. conducted a retrospective analysis of the Action to Control Cardiovascular Risk in Diabetes trail and the Veterans Affairs Diabetes trail, a total of 10 933 patients were enrolled. After 5 years of follow-up, it was discovered that even after adjusting for all other risk factors, increased BPV was still positively correlated with the risk of heart failure (40). Arnaud et al. selected 4,200 diabetic patients for an average of 6.7 years of follow-up. This research found that the highest quartile of visit-to-visit BPV can manifestly increase the risk of new-onset heart failure (HR 2.71, 95% CI 1.22–6.01) (41). Alternatively, Rossignol et al. also evaluated the impact of long-term BPV on the prognosis of patients with heart failure. They conducted a retrospective analysis of the Heart failure Endpoint evaluation of Angiotensin II Antagonist Losartan trail. A total of 3 834 patients with heart failure were recruited in the study. All patients underwent an average of 12 office blood pressure measurements after enrollment, with a median follow-up of 6.8 years. After adjusting the variables, multivariate analysis showed that the higher the visit-to-visit BPV in patients with heart failure, the worse the prognosis (HR 1.023, 95% CI 1.013–1.034) (42). The mechanisms of abnormal BPV leading to the occurrence and exacerbation of heart failure may be because on the one hand patients with increased BPV exist more active sympathetic nerve function, which activates the renin-angiotensin-aldosterone system, resulting in the increment in synthesis and release of aldosterone, and then inducing pathological changes of the cardiac structure (43). On the other hand, it may be related to inflammation. In addition to the impact on coronary artery disease and heart failure, empirical work has suggested that abnormal BPV can independently predict the occurrence of arrhythmia. A Korean study of 8 063 922 middle-aged patients with hypertension indicated that during the 7 years of follow-up, patients with the highest quartile of visit-to-visit systolic BPV is at a higher risk of new-onset atrial fibrillation than those with the lowest quartile (HR 1.06, 95% CI 1.05–1.08) (44). Lee et al. included 6 819 829 healthy individuals. After 6 years of follow-up, they found that the higher the visit-to-visit systolic BPV, the greater the danger of developing atrial fibrillation (HR 1.110, 95% CI 1.076–1.144) (45). The mechanism of arrhythmia caused by high BPV may be due to the increment in sympathetic nervous tension which promotes the secretion of catecholamines, while the attenuate vagus nerve tone dampens the secretion of acetylcholine. This change in neurohumoral regulation can affect the action potential of cardiomyocytes by exciting the corresponding ion channels, thereby altering its electrophysiological characteristics, causing reentry or triggering events, thereby inducing the occurrence of arrhythmia (46).

## The Effects of BPV on Coronary Plaques

The formation of coronary atherosclerotic plaques can cause stenosis of the coronary arteries, and the composition and morphology of coronary atherosclerotic plaques are the decisive factors for 80% of adverse coronary events (47). Through autopsy and intracoronary imaging technologies such as optical coherence tomography (OCT) and intravenous ultrasound (IVUS), a remarkable breakthrough has been made in the identification of plaque morphology and composition. Pathologically, the thrombus is directly connected with the larger necrotic lipid core through the impaired areas, which disrupts the continuity of the plaque surface (48). In addition, due to the reduction in smooth muscle cells and extracellular matrix, the fibrous cap of plaques become thinner at the lesion site, and the surrounding area is often accompanied by infiltration of inflammatory cells, such as macrophages, activated T lymphocytes, and dendritic cells (49, 50). The other type of thrombus triggered by PE is usually attached to an irregular endothelial surface (51). Through the serial sections of the artery segment, the continuity of the plaque surface is intact and endothelial cells are denuded (52). PE usually lacks a large lipid core, and inflammatory cell infiltration is also mild. Abundant proliferating smooth muscle cells, proteoglycans, and hyaluronic acid, as well as a body of new blood vessels, can be viewed around it (48, 53). Based on imaging, almost 100% of PRs are unstable lipid plaques, and the thrombus burden of PR is more serious. However, more than half of the PEs are fibrous plaques with thicker fibrous caps and smaller lipid cores, so these plaques are relatively stable (3). Clinical studies show that PR is more associated with traditional coronary risk factors (hypertension, diabetes mellitus, and dyslipidemia) than PE (54, 55). Moreover, acute coronary events caused by PR have a higher risk of large-scale infarction and absence of reflow than those caused by PE (56). Abnormal BPV can lead to the occurrence and exacerbation of CHD, however, its mechanism is not clear. Donald et al. suggested that this may be related to the increase in BPV, leading to aggravation of atherosclerosis. In their study, they found that the standardized β (95% CI) for the relationship between increased BPV and the percentage of atherosclerotic volume was 0.096 (0.026–0.166), which means that the degree of atherosclerosis can be further expanded with the increase in BPV. That study clarified the relationship between BPV and plaque formation, but did not further explore the effect of BPV on plaque composition (32). Subsequently, Aoyama et al. used OCT to analyze plaque components and calculated BPV using blood pressure values taken at baseline and during several follow-ups from 36 patients with stable angina. They highlighted that the lipid arc of the plaques is significantly related to all common quantitative parameters of long-term systolic BPV (57). Kato et al. used integrated backscatter IVUS in 102 patients with CHD. They once again verified that increased BPV can increase the volume of atherosclerosis. Moreover, they also found that in the group with higher variability of visit-to-visit systolic blood pressure, the percentage of lipid volume was higher (β = 0.56), while the fibrous plaque volume was smaller (58). Consequently, when blood pressure fluctuates, it is more likely to cause the plaques to become unstable or even rupture, resulting in MACE.

## The Mechanisms of The Impact of BPV on the Formation of Coronary Plaques

### Inflammation

It has been recognized that atherosclerosis is an inflammatory disease. Inflammation is involved in the pathological process of atherosclerosis (e.g., deterioration, exudation, hyperplasia) (59). When the body is exposed to external stimuli or damage, it releases a large number of pro- and anti-inflammatory factors to mediate and contain inflammatory reactions. Once the balance between pro-inflammatory and anti-inflammatory factors is disrupted, various inflammatory mediators and inflammatory cells promote the occurrence and development of atherosclerosis and accelerate plaque formation, which in turn induces a series of adverse coronary events (60–62). Studies have shown that abnormal BPV can upregulate the expression of C-reactive protein (CRP) (63, 64). On one hand, elevated CRP can induce vascular endothelial cells to secrete chemotactic factors and cell adhesion molecules, which can promote the migration of monocytes to the subendothelial layer of blood vessels to transform into macrophages to take up lipids and promote the transformation and proliferation of smooth muscle cells (65, 66). On the other hand, it can also cause lipid deposition by activating the complement system, affecting endothelial function and enhancing phagocytosis by promoting the activation of monocyte-macrophages to form a large number of foam cells, thereby promoting plaque formation (67). In addition, increased BPV can also promote the secretion of tumor necrosis factor-α (TNF-α) from monocytes and macrophages to increase the transport of low-density lipoproteins across endothelial cells and accelerate the process of early atherosclerosis (68, 69). BPV can also cause the accumulation of lipids by increasing TNF-α levels to destroy vascular endothelial cells and inhibit protease activity, thereby reducing lipid degradation efficiency, accelerating plaque formation, and further inducing vascular endothelial cell apoptosis to cause PR (70, 71). Recently, Julieta et al. evaluated the protective effects of new-generation and traditional β-blockers on the cardiovascular system (72). In that study, they found that stabilizing BPV can reduce the production of interleukin-6 (IL-6). This may weaken the role of IL-6 in accelerating lipid deposition, inducing hepatocytes to synthesize CRP, and stimulating the synthesis of a large number of matrix metalloproteinases to promote PR (73). It can be concluded from the above that BPV can aggravate atherosclerosis and induce PR by intervening in the production and expression of inflammatory mediators.

### Hemodynamics

Vascular endothelial cells respond uniquely to hemodynamic changes, and can convert mechanical stimulation into intracellular signals to influence cell function and gene expression, consequently affecting the pathological process of atherosclerotic plaque formation (74). The wall shear stress is a kind of parallel friction force exerted by blood flow on the vascular endothelial cell layer. Its value and direction can be constantly varied. There is a growing body of researches have pointed out that low and oscillating wall shear stress can not only promote the progression of atherosclerosis, but is also a powerful stimulating factor leading to plaque vulnerability. Because it can mediate the deposition of lipids and the regeneration of nourishing blood vessels in the plaques (48, 75, 76). Abnormal fluctuations of blood pressure can affect the shear stress acting on the surface of endothelial cells or plaques. Xiong et al. simulated the hemodynamics of stenotic arteries to quantify the effect of different degrees of BPV on the stenotic lumen. They observed that the model with the largest BPV fluctuation had the fastest blood flow velocity and the highest oscillation shear stress (OSS) compared with the stable BPV model (77). First, shear stress oscillations caused by blood pressure fluctuations can expedite endothelial dysfunction by influencing ATP-gated purinergic ligand-gated ion channel 7 receptors to integrate vascular mechanical responses with purinergic transduction (78). Second, OSS-induced endothelial dysfunction can also be achieved by enhancing the expression and nuclear accumulation of histone deacetylases I and II, respectively, as well as by regulating DNA methylation, which is related to endothelial gene expression and atherosclerosis (79, 80). Furthermore, Piezo1 and Piezo2 are multi-channel transmembrane proteins that play a key role in the regulation of cardiovascular development and physiological functions. Under physiological conditions, Piezo1 can exert anti-atherosclerotic effects by regulating nitric oxide (NO) released by endothelial cells (81). When the blood pressure fluctuates so widely that the OSS acting on the endothelial cells exceeds the normal range, it will affect the activated Piezo1 to advance the formation and progression of atherosclerotic plaques through the NF-κB pathway (82). As mentioned above, oscillations in the shear force caused by BPV can regulate endothelial cell function through the pathways of epigenetic or mechanically sensitive cation channels, thereby affecting the pathological process of atherosclerotic plaques.

### Vascular Smooth Muscle Cells

The proliferation and apoptosis of vascular smooth muscle cells (VSMCs) play a central role in the formation and progression of atherosclerotic plaques. In the early stage of atherosclerotic plaque formation, VSMCs change from a contractile to a synthetic phenotype. The synthetic phenotype of VSMCs is characterized by the decreased expression of contractile proteins. Thus, it enhances the expression of growth factors, receptors, and extracellular matrix metalloproteinases, helping VSMCs migrate and proliferate from the media to the intima to form plaques (83). In the middle stage of atherosclerotic plaque formation, VSMCs transform into the macrophage phenotype, thus acquiring the properties of macrophages, such as reducing the ability to remove lipids and dead cells to aggravate inflammation and further promote the progression of plaques (84). In the late stage of atherosclerotic plaque formation, VSMCs are continuously apoptotic and release a large amount of matrix metalloproteinases to degrade the extracellular matrix, which leads to the thinning of the fibrous cap of plaques. Alternatively, smooth muscle cells can release a large amount of lipids that have been absorbed after apoptosis, which increases the lipid burden in the plaque, thereby decreasing plaque stability and making it easier to rupture (85). When the continuity of the plaque surface is broken, under the influence of coagulation factors V and VII, apoptotic VSMCs can directly generate thrombus through phosphatidylserine that is exposed on its surface (86). Subsequently, acute cardiovascular complications occur. Researches have shown that abnormal BPV can affect the VSMCs. Aoki et al. performed bilateral sinoaortic denervation in spontaneously hypertensive rats and discovered that increased BPV can accelerate the proliferation and migration of VSMCs by chronically stimulating angiotensin-II derived from the renin-angiotensin system (87). Suki et al. simulated different degrees of BPV acting on VSMCs and indicated that an increase in blood pressure fluctuations can induce apoptosis of VSMCs by mechanically stretching the cell membrane and altering the G protein conformation (88). Therefore, BPV can affect the structure and function of VSMCs through a variety of pathways and active substances to accelerate the formation and destruction of plaques.

### Vascular Endothelial Cells

**Figure 1:**
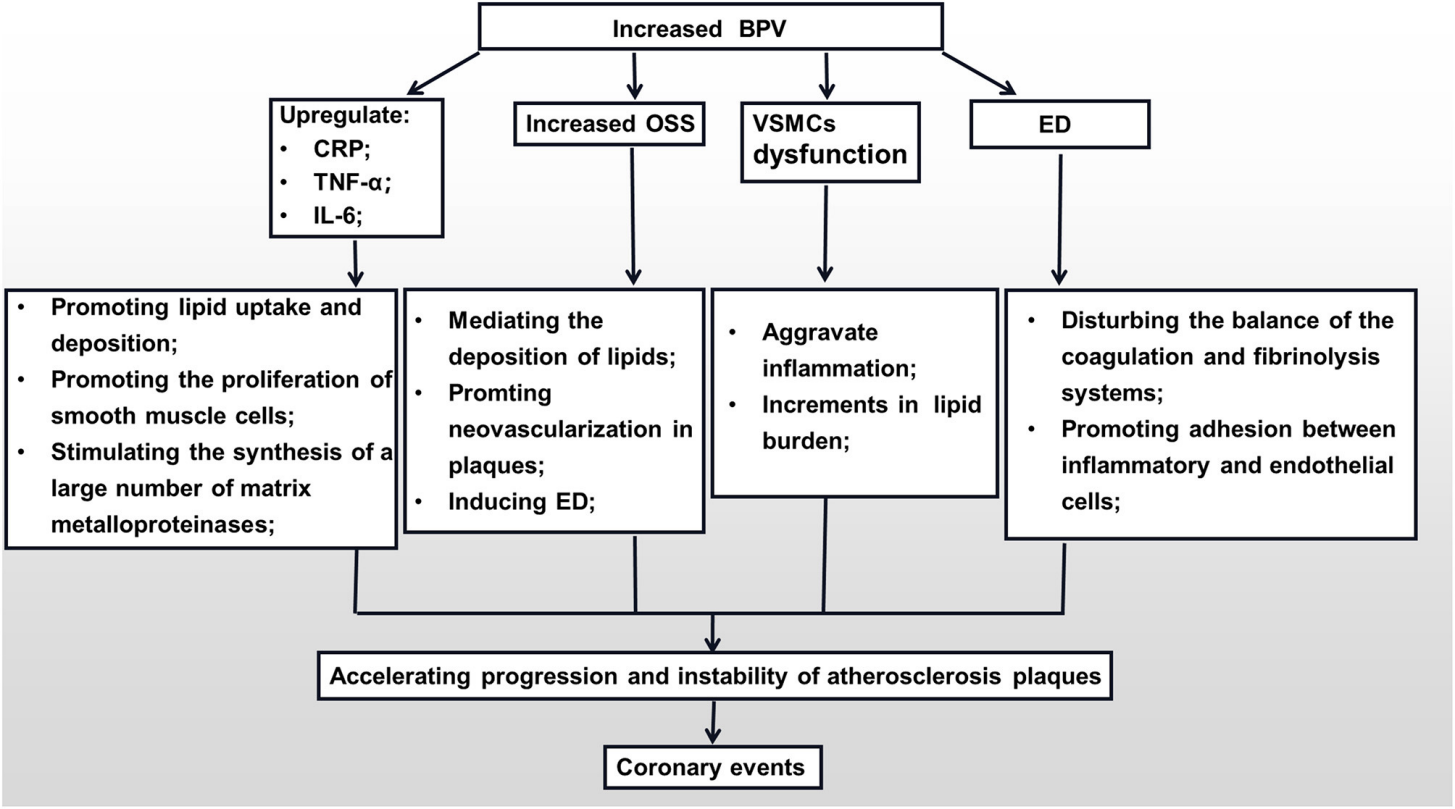
The mechanisms of the impact of BPV on the formation of coronary plaques. BPV, blood pressure variability; CRP, C-reactive protein; TNF-α, tumor necrosis factor-α; IL-6, interleukin-6; OSS, oscillation shear stress; VSMC, vascular smooth muscle cell; ED, endothelial dysfunction.

Endothelial cells are blood vessels' mechanical protective barriers. Under physiological conditions, they can secrete a variety of vasoactive substances to regulate the vasomotor state, maintain the balance of the coagulation and fibrinolysis systems, inhibit platelet aggregation, and prevent adhesion between inflammatory and endothelial cells (89). However, under pathological conditions, vascular endothelial dysfunction is not only clearly associated with CHD but is also an independent predictor of the increase in the rate of future cardiovascular events in CHD patients (90). Large quantities of empirical studies have confirmed that, on one hand, abnormal BPV is closely related to the endothelial dysfunction (ED) (91, 92). Increased BPV can lead to decreased synthesis and faster degradation of vascular endothelial-derived relaxing factors (NO, prostacyclin), and can promote the overexpression of endothelial-derived contractile factor endothelin-1 (ET-1) (93, 94). Both these effects can lead to dysfunction of vascular endothelial cells, affecting the regulation of vascular tone, decreasing the adhesion of platelets and white blood cells, and altering the expression of anticoagulants. On the other hand, with the increase in BPV, the production of NO will be reduced, and the levels of ET-1 and angiotensin-II will increase, which will cause the coronary arteries to contract severely and reduce the coronary blood flow (95). Finally, under the combined action of ED and local inflammation caused by BPV, the stability of coronary plaques is destroyed, which further leads to adverse CVDs (Figure 1).

## Conclusion

Although there is sufficient evidence that abnormal BPV is closely related to adverse CVDs, but clinical work did not use BPV as a therapeutic target. This may be due to the lack of definite indicators to distinguish pathological and physiological BPV, or the lack of a standardized assessment method of BPV, A full understanding of the relationship between BPV and coronary atherosclerotic plaques can better formulate relevant treatment strategies based on the pathogenesis and take preventive intervention measures on time. Thus, in the future, further pathophysiological experiments, clinical studies, and *in vivo* imaging studies are required for in-depth exploration. We hope this increased knowledge will help reduce the occurrence of acute cardiovascular events and improve survival rates.

## Author Contributions

HJ and BY designed the framework and direction of the manuscript. YL and XL wrote the manuscript. All authors approved the final manuscript.

## Funding

This research was supported by the National Natural Science Foundation of China (No. 82061130223 and No. 82072031), HMU Marshal Initiative Funding (HMUMIF-2016) and Fund of Key Laboratory of Myocardial Ischemia Ministry of Education (KF202020/LX).

## Conflict of Interest

The authors declare that the research was conducted in the absence of any commercial or financial relationships that could be construed as a potential conflict of interest.

## Publisher's Note

All claims expressed in this article are solely those of the authors and do not necessarily represent those of their affiliated organizations, or those of the publisher, the editors and the reviewers. Any product that may be evaluated in this article, or claim that may be made by its manufacturer, is not guaranteed or endorsed by the publisher.
